# Thrombospondin-1 in a Murine Model of Colorectal Carcinogenesis

**DOI:** 10.1371/journal.pone.0139918

**Published:** 2015-10-13

**Authors:** Zenaida P. Lopez-Dee, Sridar V. Chittur, Hiral Patel, Aleona Chinikaylo, Brittany Lippert, Bhumi Patel, Jack Lawler, Linda S. Gutierrez

**Affiliations:** 1 Department of Biology, Wilkes University, Wilkes Barre, Pennsylvania, United States of America; 2 Center for Functional Genomics, University of Albany, State University of New York, Renssaeler, New York, United States of America; 3 Department of Pathology, Beth Israel Deaconess Medical Center, Harvard Medical School, Boston, Massachusetts, United States of America; Jawaharlal Nehru University, INDIA

## Abstract

Colorectal Cancer (CRC) is one of the late complications observed in patients suffering from inflammatory bowel diseases (IBD). Carcinogenesis is promoted by persistent chronic inflammation occurring in IBD. Understanding the mechanisms involved is essential in order to ameliorate inflammation and prevent CRC. Thrombospondin 1 (TSP-1) is a multidomain glycoprotein with important roles in angiogenesis. The effects of TSP-1 in colonic tumor formation and growth were analyzed in a model of inflammation-induced carcinogenesis. WT and TSP-1 deficient mice (TSP-1^-/-^) of the C57BL/6 strain received a single injection of azoxymethane (AOM) and multiple cycles of dextran sodium sulfate (DSS) to induce chronic inflammation-related cancers. Proliferation and angiogenesis were histologically analyzed in tumors. The intestinal transcriptome was also analyzed using a gene microarray approach. When the area containing tumors was compared with the entire colonic area of each mouse, the tumor burden was decreased in AOM/DSS-treated TSP-1^-/-^ versus wild type (WT) mice. However, these lesions displayed more angiogenesis and proliferation rates when compared with the WT tumors. AOM-DSS treatment of TSP-1^-/-^ mice resulted in significant deregulation of genes involved in transcription, canonical Wnt signaling, transport, defense response, regulation of epithelial cell proliferation and metabolism. Microarray analyses of these tumors showed down-regulation of 18 microRNAs in TSP-1^-/-^ tumors. These results contribute new insights on the controversial role of TSP-1 in cancer and offer a better understanding of the genetics and pathogenesis of CRC.

## Introduction

Thrombospondins (TSP-1 through -5) are multimodular glycoproteins secreted into the extracellular matrix. TSP-1 (also called THBS1) is a 450 kDA protein recognized as an inhibitor of angiogenesis. It plays a vital role in development, inflammation and cancer as well. Studies have shown that TSP-1 inhibits cell proliferation and induces apoptosis. It is well known that some of the anti-angiogenic functions of TSP-1 are carried out by its interaction with the receptor CD36. By this mechanism, TSP-1 is able to inhibit VEGF-A and down regulate VEGFR2 phosphorylation [1,2]. The suppression of VEGFR2 phosphorylation is accomplished by the binding of SHP-1 (SRC homology 2 domain containing protein tyrosine phosphatase 1) with the VEGFR2/CD36 signaling complex [3]. TSP-1 also inhibits angiogenesis by interacting with nitric oxide in endothelial and vascular smooth muscle cells [4]. The inhibition of angiogenesis is just one of the mechanisms by which TSP-1 may suppress tumorigenesis [5]. It has been reported that Kras can interact with TSP-1 in lung cancers in a p53-dependent manner. TSP-1 can stabilize p53 by interacting directly with ERK [6]. TSP-1 binds and activates TGFβ1, regulating cytokine response and secretion of other growth factors [7].

Several studies, however, have shown that TSP-1 promotes angiogenesis [8] and favors cancer progression [9]. These contradictory results suggest that the biological activities of TSP-1 could depend on the conformation and concentration of TSP-1, which may be dependent on the tumor-type [10].

The expression of TSP-1 in CRC seems to be ambiguous. TSP-1 expression in human CRC was not different than the observed in normal colons in one study [11]. In contrast with these results, its expression was found correlated with decreased angiogenesis and good prognosis in CRC [12]. TSP-1 expression could be regulated in a post-transcriptional manner by microRNAs (miRNAs). As an example, inhibition of TSP-1 by miR-194 promoted angiogenesis and tumor growth of colonic carcinoma xenografts [13].

Apc^Min^ mice with a deficiency of TSP-1 showed an increase in adenoma numbers and developed earlier carcinomas when compared with Apc^Min^ mice controls. Interestingly, no differences in tumor vascular density were found between these mice and their control littermates [14]. The sequence adenoma-carcinoma on sporadic human CRC differs greatly from cancers originated from the transition from chronic inflammation to dysplasia-carcinoma.

Elevated levels of TSP-1 have been detected in experimental models of colitis and patients affected with inflammatory bowel disease (IBD) [15], [16]. IBD includes ulcerative colitis and Crohn’s disease [17]. These idiopathic diseases seriously diminish the quality of life of afflicted individuals and significantly increase the risk for colorectal cancer.

The treatment with azoxymethane (AOM) combined with DSS is a well-established model for the study of colorectal carcinogenesis resulting from chronic inflammation as it occurs in IBD [18]. DSS causes inflammation and induces colitis while the carcinogen AOM increases the probability that the inflammation will progress into cancer [19]. The gene expression profile [20] and the protein profile [21] of AOM-DSS treated WT mice have been reported. In addition, a reference gene expression dataset for normal human colonic epithelium is available for use in comparisons of diseased or neoplastic tissues in colon-related studies [22]. Results of a previous study using TSP-1^-/-^ mice treated with DSS showed changes suggestive of a more intense colitis. TSP-1^-/-^ mice displayed severe signs of rectal bleeding, a higher level of crypt damage, deeper lesions, as well as enhanced inflammation and angiogenesis compared to the WT controls [23,24]. Peptides derived from the type 1 repeats of TSP-1 have been used as treatment for abating the inflammatory response in mice with induced colitis [25]. These results indicated that particular sequences of TSP-1 might have specific effects in the inflammatory response and angiogenesis in the DSS model.

The present study aims to better elucidate the role of TSP-1 in carcinogenesis induced by inflammation. Tumor burden in AOM/DSS treated mice was analyzed. The morphological features of TSP-1^-/-^ tumors as well as their proliferation status and angiogenic potential were examined. In addition, a gene microarray approach was used to analyze the transcriptional profile of TSP-1^-/-^ tumors and compared with the transcriptional profile of TSP-1^-/-^ normal untreated colons. The results herein indicate that the lack of TSP-1 actually reduced the tumor burden but TSP-1^-/-^ tumors showed more angiogenesis and higher proliferation rates. Gene transcripts that might contribute to these results are discussed as well. The results shown herein indicate that TSP-1 could significantly regulate tumorigenesis in a temporal-spatial manner, promoting angiogenesis and proliferation once tumors are fully developed.

## Materials and Methods

### Animals and treatments

All the animal procedures were performed following the U.S. National Institutes of Health (NIH) guidelines and with the approval of the Wilkes University Institutional Animal Care and Use Committee (Wilkes IACUC protocol # 189).

Seven week-old male WT and TSP-1^-/-^ mice of the C57BL/6 strain (purchased from The Jackson Laboratory, Bar Harbor, ME) were used in this study. DSS with a molecular weight of 36,000–50,000, (MP Biomedical, LLC, Aurora, OH) was dissolved in the drinking water (distilled) of WT (n = 46) and TSP-1^-/-^ (n = 56) mice at a dilution of 1.5% (wt/vol) and administered to 7-week old mice for four cycles, each lasting 7 days, to induce colitis. Mice were given plain water for two weeks between each DSS cycle. A single intraperitoneal injection (10 mg/kg) of the carcinogen AOM was given to the same mice one week before the first DSS cycle. To evaluate only tumors and not inflammatory pseudopolyps, mice were sacrificed 4 weeks after the last DSS treatment. All the treated mice were sacrificed 15 weeks after the AOM injection. AOM/DSS treated mice, as well as WT (n = 7) and TSP-1^-/-^ (n = 7) untreated control mice were sacrificed by CO_2_ asphyxiation.

### Tumor and dysplasia quantification

Intestines were removed, opened longitudinally and rinsed with ice-cold phosphate buffer solution (PBS) then fixed overnight in Histochoice MB (Electron Microscopy Sciences, Hatfield, PA). Tissues were transferred to 15 ml tubes and coded. Grossly visible tumors were counted and diameters measured with a caliper. Tumor area and total colon area were measured and the percentage of tumor area per total colon area was calculated. Evaluations were performed without any knowledge of the genotype of the mice or type of treatment. Colonic tissues were processed, sectioned and stained with hematoxylin and eosin (H&E) for histological evaluations.

### Histology and inflammation grade analyses

Sections were stained with H&E for histopathological analysis. The entire colon was analyzed for dysplasia; the presence of dysplasia was confirmed under high-power magnification, ×400. The number of fields of vision was counted, and the percentage with dysplasia was calculated as the number of dysplastic segments per field. Inflammation was graded as follows: 0, no inflammation; 1, modest numbers of infiltrating leukocytes in the lamina propria; 2, infiltration of leukocytes leading to separation of crypts and mild mucosal hyperplasia; 3, massive infiltration of inflammatory cells accompanied by disrupted mucosal architecture and complete loss of goblet cells. Slides were double-coded before pictures were taken and frames blindly analyzed in a monitor.

### Immunohistochemistry (IHC)

Colon tissue sections were deparaffinized by using xylene series and hydrated through graded ethanol series (100%, 95%, 70%). After rinsing in tap water, tissue sections were incubated in a hydrogen peroxide blocking solution for 10 minutes. The sections were then washed with PBS and incubated for 1 hour in a working solution of anti M.O.M.^TM^ Mouse Ig Blocking Reagent (Vector Laboratories, Burlingame, Calif., USA). Tissue sections were again washed in PBS and then incubated in ready-to-use 2.5% normal horse or goat serum (Vector Laboratories) for 30 minutes. Sections were then incubated overnight with the following primary antibodies: WIF-1, SSTR-1 (somatostatin receptor 1), group II PLA2g2 (phospholipase A2) and CD31 (Santa Cruz Biotechnologies, Santa Cruz, Calif., USA), neurotensin (Novus Biologicals, Littleton, CO), PCNA (proliferating cell nuclear antigen) and MECA-32 (Biolegend, San Diego, CA). Sections were then washed with PBS the next day and incubated for 30 minutes in the anti-rabbit, anti-rat or anti-mouse M.O.M.^TM^ ImmPRESS^TM^ Reagent (Vector Laboratories). Tissues were again washed in PBS and then incubated in a peroxidase substrate solution, ImmPACT^TM^ DAB (Vector Laboratories) as chromogen.

### Angiogenesis and proliferation analyses

Colonic sections stained with antibodies against MECA-32 and CD31 were first scanned at low magnification to identify tumors. Multiple pictures were taken covering the entire area of the tumors at x400 magnification. Pictures were coded and counting of blood vessels was performed by multiple observers using the Leica application suite (LAS) V3.7 system. Sections stained with PCNA were also screened for tumors and each tumor area photographed and coded for further analyses. Pictures were blindly analyzed and the rate of PCNA positive cells was calculated as the number of positive PCNA nuclei over the total number of cells in each picture.

### Microarray experiments

Total RNA isolation and processing for microarray hybridization were as previously described [25]. RNA from normal and diseased colonic tissues of at least three mice from the AOM-DSS treated (TSP-1^-/-^ and WT) and three mice each from untreated TSP-1^-/-^ and WT were submitted to the Center for Functional Genomics, University of Albany, Rensselaer, New York, for microarray processing and statistical analysis. The MIAME guidelines for microarray experiments were followed.

### Microarray Data Analysis

The signals were quantile normalized using PLIER16 algorithm and baseline transformed to the median of all 12 samples. The log2 normalized signal values were then filtered to remove entities that showed signal in the bottom 20th percentile across all samples. The list was further filtered to only include entities in which at least one out of four conditions had a coefficient of variation CV < 25.0 percent (i.e., to remove probes that are highly variable across replicates in a condition). The list was then subjected to ANOVA (p<0.05) with Benjamini Hochberg False Discovery Rate correction (p<0.05) applied. A two-fold filter was applied to identify genes that are differentially expressed between any two specific conditions. Microarray data have been deposited in the Gene Expression Omnibus (GEO) database (http://www.ncbi.nlm.nih.gov/geo/) under accession number GSE60805.

### Statistical Analysis

Data were analyzed for significance by a one-way analysis of variance (ANOVA). Calculations were performed using the Stat-View system for Macintosh (Abacus Concepts, Berkeley, CA, USA). A p value <0.05 was considered significant.

## Results

### Colons of mice deficient in TSP-1 showed fewer AOM/DSS induced tumors

**Fig 1:**
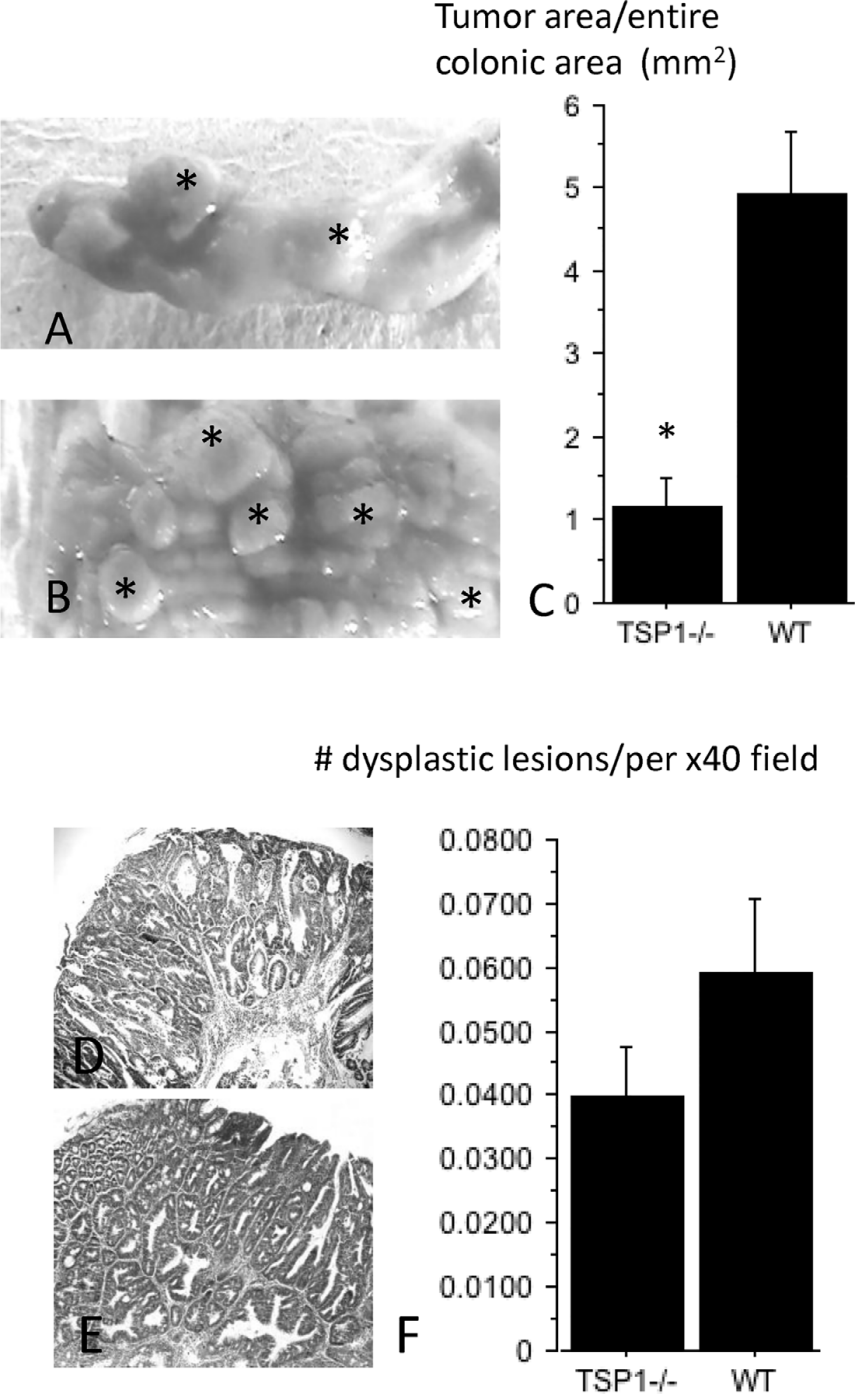
Gross and microscopic features of AOM/DSS tumors originated in WT and TSP-1^-/-^ colons. Tumors in TSP-1^-/-^ colons (A, asterisks) consisted of well-delimited polypoid lesions separated by wide areas of normal mucosa. WT tumors (B, asterisks) were grossly characterized by multiple coalescing tumors delimited by little intervening normal mucosa When the area containing tumors was compared with the entire colonic area of each mouse, the tumor burden was decreased in AOM/DSS-treated TSP-1^-/-^ versus WT mice (p = 0.0060), (C). All the microscopic dysplastic foci were analyzed in TSP-1^-/-^ (D) and WT colonic sections (E). No significant differences were observed in the number of dysplasic lesions detected by microscopic field (p = 0.14) (F).

Lesions in the WT mice were characterized by multiple coalesced tumors flanked by small areas of intervening normal mucosa. However, grossly detected tumors in TSP-1^-/-^ colons consisted of isolated polyp-like lesions separated by wide areas of normal mucosa, (Fig 1A and 1B). When the area containing tumors was compared with the entire colonic area of each mouse, the tumor burden was decreased in AOM/DSS-treated TSP-1^-/-^ versus WT mice (p = 0.0060), (Fig 1C). Tumor areas developed in TSP-1^-/-^ mice showed an average area size of 1.71 mm^2^, whereas coalesced WT tumor areas measured an average of 3.45 mm^2^. However, no significant differences were detected when the diameters of each tumor were compared between the two groups (p = 0.9602). Microscopic dysplastic foci were analyzed in TSP-1^-/-^ and WT colons (Fig 1D and 1E, respectively). No significant differences were observed in the number of dysplastic lesions per field when the entire colon was evaluated by histology (p = 0.1486, Fig 1F). In addition, the inflammation grades in the colons were not significantly different between the two genotypes by the end of the study (p = 0.4092). These results suggest that endogenous TSP-1 does not protect against the initial mutational effects of AOM.

### Tumors of TSP-1 deficient mice had higher proliferation rates and more microvessels

**Fig 2:**
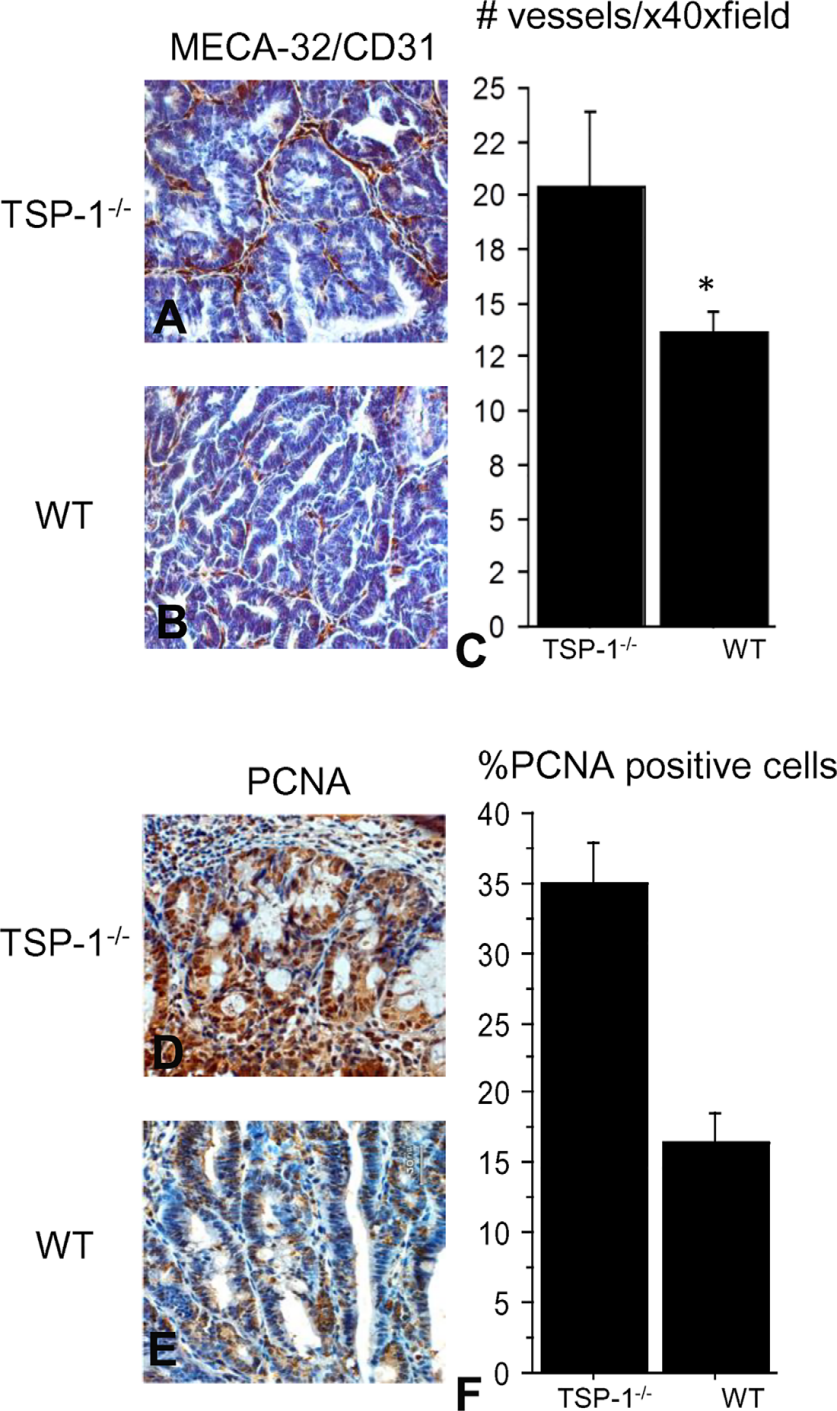
Analysis of microvascular density (MVD) and proliferation in TSP-1^-/-^ and WT tumors. Blood vessels were blindly evaluated in each tumor section stained with MECA and CD31 antibodies in TSP-1^-/-^ (A) and WT colons (B). Tumors lacking TSP-1 showed significantly higher MVD as compared to the MVD in the WT tumors (p = 0.0159), (C). IHC for PCNA was performed and positive nuclei were counted in the tumor sections of TSP-1 deficient mice (D) and WT mice (E). PCNA rates were significantly higher in tumors of TSP-1^-/-^ mice compared to WT tumors (p<.0001), (F).

Blood vessels were blindly evaluated in each tumor section stained with MECA and CD31 antibodies in TSP-1 deficient (Fig 2A) and WT colons (Fig 2B). Tumors lacking TSP-1 showed significantly higher number of microvessels as compared to the WT mice (Fig 2C) (p = 0.0159). IHC for PCNA was performed and positive nuclei were counted in the tumor sections of TSP-1-deficient mice (Fig 2D) and WT mice (Fig 2E). PCNA rates were significantly higher in tumors of TSP-1^-/-^ mice compared to WT tumors (p<.0001), (Fig 2F). Proliferation and vascular density were analyzed only in fully developed polypoid tumors.

### A set of novel genes were deregulated in the AOM/DSS treated TSP-1 deficient mice

**Fig 3:**
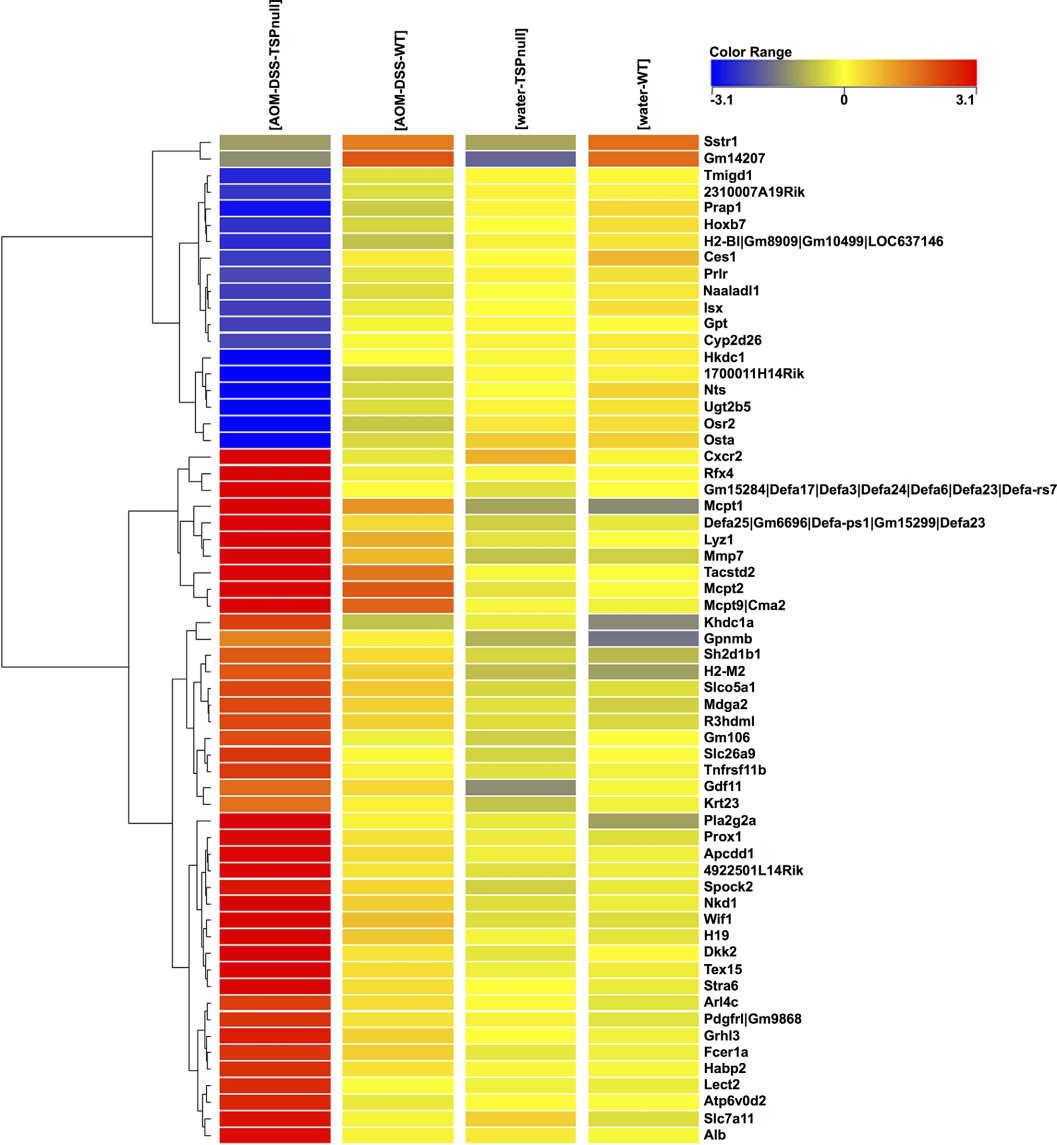
Heatmap of the 61 highly deregulated genes in the AOM-DSS treated mice. A fold-change cutoff of > = 2.0 was used and 342 differentially expressed genes in the treated (tumors) vs. untreated TSP-1^-/-^ (colonic tissue) were detected. In comparing TSP-1^-/-^ to WT tumors, 183 genes were differentially expressed. At a fold-change cutoff of > = 5.0, for the four pairwise comparison, 42 genes showed increased expression and 19 showed lowered transcript levels (p-value < 0.05). Rfx4 (regulatory factor X, 4) and Osr2 (odd-skipped related 2) showed the highest differential expression in the treated groups, TSP-1^-/-^ vs. WT, with Rfx4 expression enhanced by FC = 83.3 and Osr2 expression reduced by FC = 13.3. Transcripts from inhibitors of the Wnt signaling pathway such as WIF1 and Apcdd1 were upregulated in TSP-1^-/-^ tumors. Conversely, transcripts with known neuroendocrine-related functions such as SSTR2 and NST were downregulated in TSP-1^-/-^ tumors.

The treatment of TSP-1^-/-^ mice with AOM-DSS resulted in significant deregulation of genes involved in cell transcription, canonical Wnt signaling, transport, defense response, regulation of epithelial cell proliferation and metabolism (Table 1). A fold-change cutoff of > = 2.0 was used and 342 differentially expressed genes in the treated vs. untreated TSP-1^-/-^ were detected. In comparing treated TSP-1^-/-^ to treated WT, 183 genes were differentially expressed. At a fold-change cutoff of > = 5.0, for the four pairwise comparison, 42 genes showed increased expression and 19 showed lowered transcript levels (p-value < 0.05), as shown in the heatmap (Fig 3). Rfx4 (regulatory factor X, 4) and Osr2 (odd-skipped related 2) showed the highest differential expression in the treated groups, TSP-1^-/-^ vs. WT, with Rfx4 expression enhanced by FC = 83.3 and Osr2 expression reduced by FC = 13.3 (S1 Table). In addition, two down-regulated genes were uniquely found in the TSP-1^-/-^mice when compared to WT (in both treated and untreated pairs): Somatostatin receptor 1 (Sstr1) and Predicted gene 14207 (Gm14207) (Table 1).

**Table 1:** The highly deregulated genes, up-regulated (left column) and down-regulated (right column), in the TSP-1 deficient mice after AOM-DSS treatment are categorized according to molecular function/ontology, below. Numbers in brackets represent fold-change: first value refers to fold-change in the comparison between treated TSP-1^-/-^ vs water-TSP-1^-/-^; second value refers to fold-change comparing treated TSP-1^-/-^ vs treated WT, p<0.05).

Up-regulated genes	Down-regulated genes
Integral to membrane	Integral to membrane
• *Tacstd2* [96.0] [29.5]	• *Tmigd1* [-6.9] [5.1]
Transcription regulation	Transcription regulation
• *Rfx4* [77.1] [83.3]	• *Hoxb7* [-5.8] [-4.2]
	• *Isx* [-5.0] [-4.3]
Transport // receptor activity	Transporter activity
• *Atp6v0d2* [6.1] [7.6]	• *Osta* [-22.0] [10.7]
• *Slc7a11* [5.1] [8.2]	Neuropeptide hormone activity//blood vessel size regulation
• *Stra6* [10.8] [8.3]	• *Nts* [-10.5] [-7.8]
Defense response to bacterium	Glycolysis // hexokinase / phosphotransferase activity
• *Defa17* [53.1] [42.4]	• *Ugt2b5* [-13.4] [-9.4]
• *Defa25* [37.3] [19.1]	• *Hkdc1* [-8.0] [-8.2]
• *Lyz1* [21.7] [8.9]	cAMP-dependent protein kinase regulator activity
Proteolysis	• *2310007A19Rik* [-6.2] [-4.1]
• *Mcpt1* [49.2] [9.5]	Transaminase activity // pyridoxal phosphate binding
• *Mcpt2* [45.1] [9.2]	• *Gpt* [-5.3] [-4.6]
• *Cma2* [35.8] [9.1]	Extracellular component
• *Mmp7* [29.7] [10.0]	• *Prap1* [-8.2] [-4.9]
Negative regulation of epithelial cell proliferation	Positive regulation of cell proliferation
• *Pla2g2a* [14.3] [10.8]	• *Osr2* [-25.4] [-13.3]
• *H19* [10.9] [6.3]	Hydrolase activity
G-protein coupled signaling	• *Ces1* [-5.2] [-6.2]
• *Cxcr2* [10.3] [23.9]	Signal transduction
Apoptosis	• *Sstr1* [–––] [-6.6]
• *Khdc1a* [5.8] [8.3]	Protection from natural killer cell mediated cytoxicity
Negative regulation of Wnt signaling	• *H2-Bl* [-6.8] [-3.6]
• *Wif1* [14.4] [6.3]	(No biological data available)
• *Dkk2* [12.0] [7.8]	• *Gm14207* [–––] [-10.7]
• *Nkd1* [11.5] [5.8]	• *1700011H14Rik* [-10.3] [-6.7]

AOM-DSS treatment did not affect the expression of the tumor suppressor p53 and oncogenes associated with CRC such as Jun and c-Myc. It is worth noting that TSP-1 was differentially expressed in TSP-1 deficient mice; these mice still have the gene but it contains a targeted mutation in exons 2–3 and intron 3. They are not strictly knockout mice but the protein-product is not produced.

Two members of the Ras oncogene family, Rab18 and Rab39b, were slightly up regulated (fold change of 2.1 and 2.2, respectively) in the AOM-DSS-TSP-1^-/-^ vs. water-TSP-1^-/-^ pair (S1 Table). RAS protein activator like 2 (Rasal2) and ras homolog gene family, member J (RhoJ) showed a two-fold increase in expression in the pairwise comparisons of AOM-DSS-TSP-1^-/-^ vs. water-TSP-1^-/-^ and AOM-DSS-TSP-1^-/-^ vs. AOM-DSS-WT (S1 Table).A comparison of the gene expression profiles of the WT mice, treated vs water, is presented in S2 Table.

The expression of microRNAs was also analyzed in this study (S3 Table). 10 miRNAs were significantly down-regulated in all AOM-DSS treated mice, both TSP-1^-/-^ and WT. The differentially regulated miRNAs include Mir1-1, Mir125b-2 and Mir141, which have well-known roles in cancer development.

### Validation of transcripts involved in key pathways of inflammation and cancer

**Fig 4:**
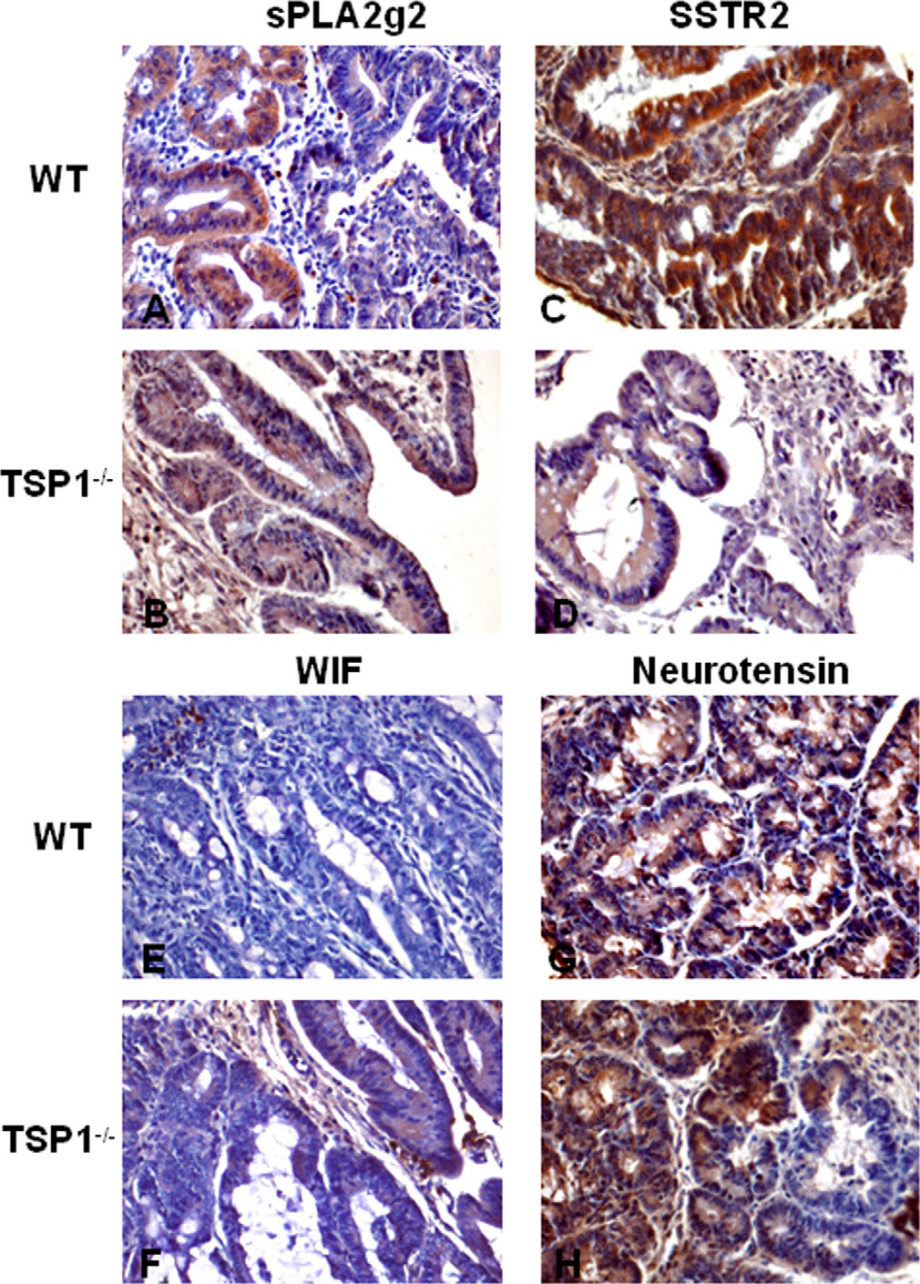
Validation of transcripts involved in inflammation and cancer by IHC. The expression of some transcripts involved in epithelial proliferation and the immune response was examined by IHC. PLA2g2 immunostaining of these tumors showed a focal localization in glands of WT tumors (A). This same protein was diffusely expressed in the glands and stroma of TSP-1^-/-^ tumors (B). SSTR2 was intensely expressed in WT tumors (C) while the staining in TSP-1^-/-^ lesions was rather light or even negative (D). WIF1 was focally located in the lamina propria of WT cancers (E). Cytoplasmic staining was detected in isolated foci of cells in malignant glands in TSP-1^-/-^ tumors (F). Neurotensin (NTS) was predominantly localized in the malignant epithelial cells. While WT tumors showed intense and diffuse staining (G), some of the tumors developed in TSP-1^-/-^ showed focal areas lacking NTS staining (H).

The expression of the proteins from some transcripts involved in mechanisms related with inflammation and cancer was examined using IHC. One of the proteins analyzed was PLA2g2. This protein has a critical role in inflammation and oxidative stress. PLA2g2 immunostaining of these tumors showed a differential localization in glands of WT tumors (Fig 4A). However this protein seemed to be more diffusely expressed in the glands and stroma of TSP-1^-/-^ tumors (Fig 4B).

SSTR2, a G-protein coupled receptor for somatostatin is an inhibitory hormone produced by immune and neuroendocrine cells [26] It is also expressed in several types of cancers, including prostate cancer and leukemia [27], [28]. SSTR2 was intensely expressed in WT tumors (Fig 4C) while the staining in TSP-1^-/-^ tumors was low or negative (Fig 4D). WIF1 is a protein encoded by the Wif gene that contains a WNT inhibitory factor. WIF1 was focally located in the lamina propria of WT cancers (Fig 4E). Cytoplasmic staining was detected in isolated malignant cells in TSP-1^-/-^ tumors (Fig 4F). Neurotensin (NTS) is a protein present in the central nervous system and intestine. High expression of neurotensin and its receptor has been detected in human and mouse colitic tissues [29]. NTS activates the growth of colon cancers and it has an important role in inflammation [30] [31] [32]. Though this transcript was significantly downregulated in TSP-1 deficient tumors (Fig 3), still these same lesions displayed increased proliferation. However, high proliferation in tumors could be solely promoted by a vigorous angiogenic response [33]. NTS expression in TSP-1 deficient tumors was predominantly localized in the malignant epithelial cells (Fig 4G). While WT tumors displayed intense and diffuse staining (Fig 4G), tumors developed in TSP-1^-/-^ colons showed focal areas negative for NTS staining (Fig 4H).

## Discussion

Numerous studies have emphasized the role of TSP-1 in cancer and metastasis. TSP-1 has anti-angiogenic properties by inducing apoptosis in endothelial cells [34]. By activating TGFß1, TSP-1 has key functions in inflammation and consequently in carcinogenesis [35]. However, TSP-1 might promote the attachment of cells to the extracellular matrix, favoring the migration of cancer cells [36]. These functions would explain the pro-tumor effects in some human cancers and animal models [37].

TSP-1 might act in different ways by interacting with multiple proteins through its several domains, thus activating different signaling pathways. Our results in this study indicate that overall tumor burden induced by the mutational action of the carcinogen AOM is significantly diminished in TSP-1 deficient colons. Areas of coalescent tumors were observed in WT colons. It has been reported that the knockdown of B-RafV600E, a mutant form of the gene BRAF, resulted in TSP-1 down-regulation in human thyroid cancer cells with a significant reduction of adhesion and migration/invasion of these cells in tumors [9].

We reported that Apc^Min^ mice with a deficiency in TSP-1 had higher numbers of polyps than controls [14]. However, Apc^Min^ mice have a mutation in the APC gene, which is rarely seen in dysplastic mucosa and neoplasias developed due colitis [38].In addition, early genomic damage by AOM in the colonic mucosa seems to be mediated through the p53 pathway [39]. Studies in the AOM/DSS model indicate that p53 is not actually mutated in these tumors but unable to activate or repress transcription [40]. Loss of p53 has been correlated to TSP-1 silencing in ovarian carcinoma, bladder cancer, glioma, prostate cancer, and renal cell carcinoma but not in gastric carcinoma [41]. In colon cancers, p53 seems to regulate TSP-1 by a posttranscriptional mechanism that involves miRNA-194 [13]. Conversely, the p53 tumor suppressor gene activates the promoter of TSP-1 [42]. TSP-1 might have a role in the nongenetic inhibition of p53 in the AOM/DSS model. Studies using mice with a deficiency in both p53 and TSP-1, resulted in fewer osteosarcomas, suggesting a strong association between these genes [5]. TSP-1 might facilitate the evasion of the pro-apoptotic mechanisms mediated by p53 in colonic cells.

The results shown herein are quite different from those observed in previous studies evaluating the roles of TSP-1 in experimental colitis. TSP-1-deficient mice displayed increased dysplasia and early cancers after multiple cycles of DSS only without AOM treatment [24]. The mutagenic effects of AOM might work by different mechanisms from the ones induced by DSS alone. As an example, mutations within codons 33 and 34 of the ß catenin gene are induced by AOM, while the mutagenic effects of DSS target codon 32 [43]. Alterations in iNOS have been detected in the AOM/DSS model, but they seem to be most likely a consequence of DSS exposure [43]. TGFß1 inhibitory mechanisms may fully function in a normal colon exposed to DSS only. AOM tumors have been reported to have defective TGFß1 activation [44]. We hypothesize that the lack of TSP-1, a major activator of this growth factor, could deplete even more the pool of active TGFß1, thus favoring AOM-induced carcinogenesis.

As tumors continue growing, the angiogenic switch is turned on inducing the formation of new blood vessels for the surveillance of proliferating malignant cells. In this study, AOM/DSS induced tumors developed in TSP-1^-/-^ colons showed higher numbers of blood microvessels and proliferation indexes despite the overall decrease in tumor burden. Similar paradoxical results showing enhanced angiogenesis with decreased tumor load have been previously reported [45]. In xenografted prostatic tumors, TSP-1 inhibited angiogenesis but it was a potent stimulator of prostate tumor cell migration [46]. In addition, the angiogenic switch might occur at later stages during the carcinogenesis process. Our results indicate that TSP-1 does not prevent AOM-tumor initiation but it could significantly delay the angiogenic switch that will promote further proliferation and metastasis. AOM tumors displayed higher numbers of PCNA positive cells in the absence of TSP-1, suggesting higher rates of proliferation. Therefore, bigger tumors would be expected. In fact, TSP-1^-/-^ tumors were usually polypoid lesions while WT displayed small and flat growing lesions. TSP-1 might enhance the migration of tumor cells and favor tumor expansion in WT tumors. As a result, WT tumors would coalesce as they grow and expand, explaining the highest tumor/colonic area index in WT colons. Spreading and migration of tumor cells could actually occur even if they display a low to moderate mitotic index. However, tumors that develop in the TSP-1 deficient colons could potentially be more malignant. In this study’s results, TSP-1 does not protect against AOM mutagenesis but it could considerably delay the angiogenic switch and further proliferation. It is possible that these AOM-induced tumors could become actual cancers at a more late stage. Further studies evaluating this model at different time points might better elucidate these paradoxical findings.

A novel contribution of this study is the detection of transcripts dysregulated by the lack of TSP-1 in the AOM/DSS model. Transcripts such as WIF1 and phospholipase A2 have been detected in gene microarray studies using this same model, validating the data herein [20]. WIF1 is increased 14.4 fold in TSP-1^-/-^ tumors compared with TSP-1^-/-^ normal colonic tissue and 6.3 times when compared with the WT tumors. In addition, a strong correlation has been found between the expression of this protein and the severity of the inflammation in biopsies from patients suffering from ulcerative colitis (manuscript in preparation). The transcription factor Rfx4, a highly upregulated transcript in TSP-1 deficient tumors, is believed to affect critical pathways in cancer such as WNT signaling [47].

TSP-1 interacts with a variety of growth factors and tumor suppressor genes such as p53, proteoglycans and TGFß signaling pathways. One of the transcripts dysregulated in our study, is Osr2, known to be a regulator of epithelial-mesenchymal interactions, tissue development and proliferation [48]. By interacting with TGFß, Osr2 reduces cell migration while stimulating cell-cycle progression. Osr2/TGFß axis induces these effects by activating the Smad3-ATF2 transcriptional complex [49].

In colonic samples from patients with IBD-related CRC, neurotensin (NTS) and its receptor NTSR1 were found to be significantly higher in epithelial cells when compared to cells of healthy tissues [50] [51]. The NTS/NTSR1 signaling pathway is involved in regulating tumorigenesis in colitis-associated dysplasia [31}{51][32]. The fact that this transcript is downregulated in TSP-1 deficient colons, which showed fewer tumors, seems to validate NTS as an important regulator of colonic carcinogenesis. However, the axis NTS/NTSR1 is a promoter of proliferation. Our studies show that these same tumors displayed higher PCNA indexes. Early *in vitro* and *in vivo* studies with squamous cell carcinomas demonstrated that TSP-1 certainly reduces proliferation and tumor growth, but these effects are indirect and a consequence of the inhibition of angiogenesis [33]. Without the inhibition of TSP-1, expression of angiogenic growth factors such as VEGF will induce a vigorous angiogenic response, promoting tumor proliferation. Another transcript significantly downregulated was PLA2G2. Plasma levels of this phospholipase are increased in several malignancies [52]. Furthermore, decreased plasma levels of PLA2G2 are correlated with longer survival in patients with cancers. The reduced tumor burden in TSP-1^-/-^ mice might be partly due to the deregulation of these transcripts.

TSP-1 interacts with proteins implicated in proliferation, DNA repair and transcriptional regulation such as PRMT6 (protein arginine methyltransferase 6). This protein associates with the TSP-1 promoter and represses the transcription of TSP-1 by regulating the methylation of histones [53]. Oncogenes such as Myc down-regulate TSP-1 [54] [55]. A Myc-activated microRNA cluster has been reported as one of the mechanisms by which this oncogene represses TSP-1 expression [56]. Src [57] and Jun [58] have also been shown to repress the transcription of TSP-1. TSP-1 gene is hypermethylated in several cancers [59] and aberrant methylation of several tumor suppressor genes has been detected in the AOM model [60].

Increasing evidence indicates that TSP-1 functions are closely regulated by miRNAs [61]. This study shows significant changes in 18 miRNAs. These RNAs are quite important in regulating transcription and posttranscriptional mechanisms. One of these miRNAs is miR-141, which is downregulated in AOM/DSS treated TSP-1^-/-^. This miRNA has been found up-regulated in nasopharyngeal and ovarian carcinomas [62] [63]. The presence of miR141 in prostate and colorectal cancers has also been correlated with metastasis and poor prognosis. Another down-regulated miRNA detected by the gene microarray analyses is miR-1. miR-1 is upregulated in human rabdomyosarcomas but downregulated in colorectal cancer [64], [63]. Long non-coded RNAs (lncRNAs) seem to play an important role in cancer as clinical studies show that they are indicators of poor prognosis and metastasis in several human cancers [65]. The lncRNA H19 is induced by c-Myc favoring the development of breast and lung cancers [66]. In addition, lncRNA H19 inhibits RB in human colorectal cancers [64].

In conclusion, this study describes for the first time the growth pattern, morphological changes, angiogenesis and proliferation status of tumors lacking TSP-1. Furthermore, the transcript profile of these tumors reveals the deregulated expression of novel genes that could explain the controversial roles of TSP-1 in cancers and inflammation.

## Supporting Information

S1 Table Differentially expressed genes in the AOM-DSS treated TSP-null mice. List of genes differentially expressed in the AOM-DSS treated TSP-null mice and the controls with a fold change of ≥ 2 and a p value of <0.05.
(XLS)

S2 Table Upregulated genes in the AOM-DSS treated WT mice vs. water treated WT mice. List of genes in the AOM-DSS treated WT mice vs. water treated with a fold changes of >2.2 and a p value cut off <0.05
(XLS)

S3 Table Differentially expressed miRNAs (18) in the AOM-DSS treated TSP-1 null mice. The p value cut off for this list was <0.05 for miRNAs differentially expressed at a fold change of >2.0.
(XLS)

## References

[1] Kazerounian S, Duquette M, Reyes M, Lawler J, Song K, Perruzzi C, et al Priming of the vascular endothelial growth factor signaling pathway by thrombospondin-1, CD-36, and spleen tyrosine kinase. Blood. 2011; 117: 4658–4666. 10.1182/blood-2010-09-305284 21378271 PMC3099580

[2] Primo L, Ferrandi C, Roca C, Marchiò S, di Blasio L, Alessio M, et al Identification of CD36 molecular features required for its in vitro angiostatic activity. Faseb j. 2005; 19: 1713–1715 16037098 10.1096/fj.05-3697fje

[3] Chu L, Ramakrishnan D, Silverstein R. Thrombospondin-1 modulates VEGF signaling via CD36 by recruiting SHP-1 to VEGFR2 complex in microvascular endothelial cells. Blood. 2013; 122: 1822–1832. 23896411 10.1182/blood-2013-01-482315 PMC3765061

[4] Isenberg J, Martin-Manso G, Maxhimer J, Roberts DD. Regulation of nitric oxide signalling by thrombospondin 1: implications for anti-angiogenic therapies. Nat Rev Cancer. 2009; 9: 182–194. 10.1038/nrc2561 19194382 PMC2796182

[5] Lawler J, Miao W, Duquette M, Bouck N, Bronson R, Hynes R Thrombospondin-1 gene expression affects survival and tumor spectrum of p53-deficient mice. Am J Pathol. 2001; 159: 1949–1956. 11696456 10.1016/S0002-9440(10)63042-8 PMC1867067

[6] Baek KH, Bhang D, Zaslavsky A, Wang LC, Vachani A, Kim CF, et al Thrombospondin-1 mediates oncogenic Ras-induced senescence in premalignant lung tumors. J Clin Invest. 2013; 123: 4375–4389 10.1172/JCI67465 24018559 PMC3784530

[7] Crawford SE, Stellmach V, Murphy-Ullrich JE, Ribeiro SM, Lawler J, Hynes RO, et al Thrombospondin-1 is a major activator of TGF-beta1 in vivo. Cell. 1989; 93: 1159–1170. 9657149 10.1016/s0092-8674(00)81460-9

[8] Nicosia RF, Tuszynski G. Matrix-bound thrombospondin promotes angiogenesis in vitro. J Cell Biol. 1994; 124: 183–193 7507491 10.1083/jcb.124.1.183 PMC2119887

[9] Nucera C, Porrello A, Antonello ZA, Mekel M, Nehs MA, Giordano TJ, et al B-Raf(V600E) and thrombospondin-1 promote thyroid cancer progression. Proc Natl Acad Sci U S A. 2010; 107: 10649–10654 10.1073/pnas.1004934107 20498063 PMC2890809

[10] Miyata Y, Sakai H. Thrombospondin-1 in urological cancer: pathological role, clinical significance, and therapeutic prospects. Int J Mol Sci. 2013; 14: 12249–12272 10.3390/ijms140612249 23749112 PMC3709784

[11] Yoshida Y, Oshika Y, Fukushima Y, Tokunaga T, Hatanaka H, Kijima H, et al Expression of angiostatic factors in colorectal cancer. Int J Oncol. 1999; 15: 1221–1225 10568831 10.3892/ijo.15.6.1221

[12] Miyanaga K, Kato Y, Nakamura T, Matsumura M, Amaya H, Horiuchi T, et al Expression and role of thrombospondin-1 in colorectal cancer. Anticancer Res. 2002; 22: 3941–3948 12553016

[13] Sundaram P, Hultine S, Smith LM, Dews M, Fox JL, Biyashev D, et al p53-responsive miR-194 inhibits thrombospondin-1 and promotes angiogenesis in colon cancers. Cancer Res. 2011; 71: 7490–7501. 10.1158/0008-5472 22028325 PMC3242824

[14] Gutierrez L, Suckow M, Lawler J, Ploplis V, Castellino FJ. Thrombospondin 1 a regulator of adenoma growth an carcinoma progression in the ApcMin/+ mouse model. Carcinogenesis. 2003; 24: 199–207. 12584168 10.1093/carcin/24.2.199

[15] Chidlow J, Langston W, Greer J, Ostanin D, Abdelbaqi M, Houghton J, et al Differential angiogenic regulation of experimental colitis. Am J Pathol. 2006; 169: 2014–2030. 17148665 10.2353/ajpath.2006.051021 PMC1762465

[16] Alkim C, Sakiz D, Alkim H, Livaoglu A, Kendir T, Demirsoy H, et al Thrombospondin-1 and VEGF in inflammatory bowel disease. Libyan J Med. 2012; 7 10.3402/ljm.v7i0.8942 PMC3269884 22299021

[17] Abraham C, Cho J. Inflammatory Bowel Disease. N Engl J Med 2009; 361;(21):2066–78. 10.1056/NEJMra0804647 19923578 PMC3491806

[18] De Robertis M, Massi E, Poeta ML, Carotti S, Morini S, Cecchetelli L, et al The AOM/DSS murine model for the study of colon carcinogenesis: From pathways to diagnosis and therapy studies. J Carcinog. 2011; 10: 9 10.4103/1477-3163.78279 21483655 PMC3072657

[19] Gao Y, Li X, Yang M, Zhao Q, Liu X, Wang G, et al Colitis-accelerated colorectal cancer and metabolic dysregulation in a mouse model. Carcinogenesis. 2013; 34: 1861–1869. 23615396 10.1093/carcin/bgt135

[20] Suzuki R, Miyamoto S, Yasui Y, Sugie S, Tanaka T. Global gene expression analysis of the mouse colonic mucosa treated with azoxymethane and dextran sodium sulfate. BMC Cancer. 2007; 7: 84 17506908 10.1186/1471-2407-7-84 PMC1890554

[21] Yasui Y, Tanaka T. Protein expression analysis of inflammation-related colon carcinogenesis. J Carcinog. 2009; 8: 9 10.4103/1477-3163.51851 19491504 PMC2699605

[22] Mojica W, Hawthorn L Normal colon epithelium: a dataset for the analysis of gene expression and alternative splicing events in colon disease. BMC Genomics. 2010; 11: 5 10.1186/1471-2164-1111-1185 20047688 PMC2823691

[23] Punekar S, Zak S, Kalter VG, Dobransky L, Punekar I, Lawler JW, et al Thrombospondin 1 and its mimetic peptide ABT-510 decrease angiogenesis and inflammation in a murine model of inflammatory bowel disease. Pathobiology. 2008; 75: 9–21. 000113790 10.1159/000113790 18334835

[24] Zak S, Treven J, Nash N, Gutierrez LS. Lack of thrombospondin-1 increases angiogenesis in a model of chronic inflammatory bowel disease. Int J Colorectal Dis. 2008; 23: 297–304. 18043928 10.1007/s00384-007-0397-5

[25] Lopez-Dee Z, Chittur SV, Patel B, Stanton R, Wakeley M, Lippert B, et al Thrombospondin-1 type 1 repeats in a model of inflammatory bowel disease: transcript profile and therapeutic effects. PLoS One. 2012; 7: 10.1371/journal.pone.0034590 PMC3318003 22509329

[26] Patel Y Somatostatin and its receptor family. Front Neuroendocrinol. 1999; 20: 157–198. 10.1006/frne.1999.0183 10433861

[27] Reubi J, Waser B, Schaer J, Markwalder R. Somatostatin receptors in human prostate and prostate cancer. Clin Endocrinol Metab. 1995; 80: 2806–2814 10.1210/jcem.80.9.7673428 7673428

[28] Talme T, Ivanoff J, Hagglund M, Van Neerven RJ, Ivanoff A, Sundqvist K. Somatostatin receptor (SSTR) expression and function in normal and leukaemic T-cells. Evidence for selective effects on adhesion to extracellular matrix components via SSTR2 and/or 3. Clin Exp Immunol. 2001; 125: 71–79. cei1577 11472428 10.1046/j.1365-2249.2001.01577.x PMC1906108

[29] Law IK, Pothoulakis C. MicroRNA-133alpha regulates neurotensin-associated colonic inflammation in colonic epithelial cells and experimental colitis. RNA Dis. 2015; 2:10.14800/rd.472 PMC4441413 26005712

[30] Müller K, Tveteraas I, Aasrum M, Ødegård J, Dawood M, Dajan iO, et al Role of protein kinase C and epidermal growth factor receptor signalling in growth stimulation by neurotensin in colon carcinoma cells. BMC Cancer. 2011; 11: 421 21961726 10.1186/1471-2407-11-421 PMC3196723

[31] Bugni JM, Rabadi LA, Jubbal K, Karagiannides I, Lawson G, Pothoulakis C. The neurotensin receptor-1 promotes tumor development in a sporadic but not an inflammation-associated mouse model of colon cancer. Int J Cancer. 2015; 130: 1798–1805. 10.1002/ijc.26208 PMC3288327 21630261

[32] Tasuta M, Iishi H, Baba M, Taniguchi H. Enhancement by neurotensin of experimental carcinogenesis induced in rat colon by azoxymethane. Br J Cancer. 1990; 62: 368–371 2206944 10.1038/bjc.1990.299 PMC1971434

[33] Streit M, Velasco P, Brown LF, Skobe M, Richard L, Riccardi L, et al Overexpression of thrombospondin-1 decreases angiogenesis and inhibits the growth of human cutaneous squamous cell carcinomas. Am J Pathol. 1999; 155: 441–452. 10433937 10.1016/S0002-9440(10)65140-1 PMC1866855

[34] Jimenez B, Volpert O, Crawford S, Febbraio M, Silverstein R, Bouck N. Signal leading to apoptosis-dependent inhibition of neovascularization by thrombospondin-1. Nat Med. 2000; 6(1): 41–48. 10613822 10.1038/71517

[35] Lopez-Dee Z, Pidcock K, Gutierrez LS. Thrombospondin-1: multiple paths to inflammation. Mediators Inflamm. 2011; 10.1155/2011/296069 21765615 PMC3134184

[36] Yee KO, Connolly CM, Duquette M, Kazerounian S, Washington R, Lawler J. The effect of thrombospondin-1 on breast cancer metastasis Breast Cancer Res Treat. 2009 114: 85–96 10.1007/s10549-008-9992-6 18409060 PMC2631620

[37] Tuszynski GP, Gasic TB, Rothman VL, Knudsen KA, Gasic GJ. Thrombospondin, a potentiator of tumor cell metastasis. Cancer Res. 1987; 47: 4130–4133 3607754

[38] Aust DE, Terdiman JP, Willenbucher RF, Chew K, Ferrell L, Florendo C, et al Altered distribution of beta-catenin, and its binding proteins E-cadherin and APC, in ulcerative colitis-related colorectal cancers. Mod Pathol. 2001; 14: 29–39. 10.1038/modpathol.3880253 11211307

[39] Kerr CA, Hines BM, Shaw JM, Dunne R, Bragg LM, Clarke J, et al Genomic homeostasis is dysregulated in favour of apoptosis in the colonic epithelium of the azoxymethane treated rat. BMC Physiol. 2013; 13:10.1186/1472-6793-13-2 PMC3561103 23343511

[40] Nambiar PR, Giardina C, Guda K, Aizu W, Raja R, Rosenberg DW Role of the alternating reading frame (P19)-p53 pathway in an in vivo murine colon tumor model. Cancer Res. 2002; 62: 3667–3674. 12097273

[41] Oue N, Matsumura S, Nakayama H, Kitadai Y, Taniyama K, Matsusaki K, et al Reduced expression of the TSP1 gene and its association with promoter hypermethylation in gastric carcinoma. Oncology. 2003; 64: 423–429 12759541 10.1159/000070302

[42] Dameron KM, Volpert OV, Tainsky MA, Bouck N. Control of angiogenesis in fibroblasts by p53 regulation of thrombospondin-1. Science. 1994; 265: 1582–1584. 7521539 10.1126/science.7521539

[43] Tanaka T, Suzuki R, Kohno H, Sugie S, Takahashi M, Wakabayashi K. Colonic adenocarcinomas rapidly induced by the combined treatment with 2-amino-1-methyl-6-phenylimidazo[4,5-b]pyridine and dextran sodium sulfate in male ICR mice possess beta-catenin gene mutations and increases immunoreactivity for beta-catenin, cyclooxygenase-2 and inducible nitric oxide synthase. Carcinogenesis. 2005; 26: 229–238 15459021 10.1093/carcin/bgh292

[44] Guda K, Claffey KP, Dong M, Nambiar PR, Rosenberg DW. Defective processing of the transforming growth factor-beta1 in azoxymethane-induced mouse colon tumors. Mol Carcinog. 2003; 37: 51–59. 12720300 10.1002/mc.10120

[45] Noguera-Troise I, Daly C, Papadopoulos NJ, Coetzee S, Boland P, Gale NW, et al Blockade of Dll4 inhibits tumour growth by promoting non-productive angiogenesis. Nature. 2006; 444: 1032–1037. 17183313 10.1038/nature05355

[46] Firlej V, Mathieu JR, Gilbert C, Lemonnier L, Nakhle J, Gallou-Kabani C, et al Thrombospondin-1 triggers cell migration and development of advanced prostate tumors. Cancer Res. 2011; 71: 7649–7658. 10.1158/0008-5472.CAN-7611-0833 22037878

[47] Zhang D, Zeldin DC, Blackshear PJ. Regulatory factor X4 variant 3: a transcription factor involved in brain development and disease. J Neurosci Res. 2007; 85: 3515–3522. 10.1002/jnr.21356 17510980 PMC2367213

[48] Lan Y, Ovitt CE, Cho ES, Maltby KM, Wang Q, Jiang R. Odd-skipped related 2 (Osr2) encodes a key intrinsic regulator of secondary palate growth and morphogenesis. Development. 2004; 131: 3207–3216. 10.1242/dev.01175 15175245

[49] Kawai S, Amano A Negative regulation of Odd-skipped related 2 by TGF-beta achieves the induction of cellular migration and the arrest of cell cycle. Biochem Biophys Res Commun. 2012; 421: 696–700. 22542937 10.1016/j.bbrc.2012.04.064

[50] Bossard C, Souaze F, Jarry A, Bezieau S, Mosnier JF, Forgez P, et al Over-expression of neurotensin high-affinity receptor 1 (NTS1) in relation with its ligand neurotensin (NT) and nuclear beta-catenin in inflammatory bowel disease-related oncogenesis. Peptides. 2007; 28: 2030–2035. 17870207 10.1016/j.peptides.2007.06.030

[51] Gui X, Liu S, Yan Y, Gao Z. Neurotensin receptor 1 overexpression in inflammatory bowel diseases and colitis-associated neoplasia. World J Gastroenterol. 2013; 19: 4504–4510. 23901225 10.3748/wjg.v19.i28.4504 PMC3725374

[52] Menschikowski M, Hagelgans A, Schuler U, Froeschke S, Rosner A, Siegert G. Plasma levels of phospholipase A2-IIA in patients with different types of malignancies: prognosis and association with inflammatory and coagulation biomarkers. Pathol Oncol Res. 2013; 19: 839–846 10.1007/s12253-013-9652-y 23722320

[53] Michaud-Levesque J, Richard S Thrombospondin-1 is a transcriptional repression target of PRMT6. J Biol Chem. 2009; 284: 21338–21346. 19509293 10.1074/jbc.M109.005322 PMC2755858

[54] Zhou L, Picard D, Ra YS, Li M, Northcott PA, Hu Y, et al Silencing of thrombospondin-1 is critical for myc-induced metastatic phenotypes in medulloblastoma. Cancer Res. 2010; 70: 8199–8210. 20876797 10.1158/0008-5472.CAN-09-4562

[55] Kaur S, Soto-Pantoja DR, Stein EV, Liu C, Elkahloun AG, Pendrak ML, et al Thrombospondin-1 signaling through CD47 inhibits self-renewal by regulating c-Myc and other stem cell transcription factors. Sci Rep. 2013; 3: 10.1038/srep01673 PMC3628113 23591719

[56] Dews M, Homayouni A, Yu D, Murphy D, Sevignani C, Wentzel E, et al Augmentation of tumor angiogenesis by a Myc-activated microRNA cluster. Nat Genet. 2006; 38: 1060–1065. 10.1038/ng1855 16878133 PMC2669546

[57] Slack JL, Bornstein P. Transformation by v-src causes transient induction followed by repression of mouse thrombospondin-1. Cell Growth Differ. 1994; 5: 1373–1380 7696186

[58] Dejong V, Degeorges A, Filleur S, Ait-Si-Ali S, Mettouchi A, Bornstein P, et al The Wilms' tumor gene product represses the transcription of thrombospondin 1 in response to overexpression of c-Jun. Oncogene. 1999; 18: 3143–3151. 10.1038/sj.onc.1202654 10340386

[59] Li Q, Ahuja N, Burger PC, Issa JP. Methylation and silencing of the Thrombospondin-1 promoter in human cancer. Oncogene. 1999; 18: 3284–3289. 10.1038/sj.onc.1202663 10359534

[60] Borinstein SC, Conerly M, Dzieciatkowski S, Biswas S, Washington MK, Trobridge P, et al Aberrant DNA methylation occurs in colon neoplasms arising in the azoxymethane colon cancer model. Mol Carcinog. 2010; 49: 94–103. 10.1002/mc.20581 19777566 PMC2875385

[61] Dogar AM, Semplicio G, Guennewig B, Hall J. Multiple microRNAs derived from chemically synthesized precursors regulate thrombospondin 1 expression. Nucleic Acid Ther. 2014; 24: 149–159. 10.1089/nat.2013.0467 24444023 PMC3962651

[62] Luo Z, Zhang L, Li Z, Li X, Li G, Yu H, et al An in silico analysis of dynamic changes in microRNA expression profiles in stepwise development of nasopharyngeal carcinoma. BMC Med Genomics. 2012; 19: 3. 10.1186/1755-8794-5-3 PMC3293045 22260379

[63] Cheng H, Zhang L, Cogdell DE, Zheng H, Schetter AJ, Nykter M, et al Circulating plasma MiR-141 is a novel biomarker for metastatic colon cancer and predicts poor prognosis. PLoS One. 2011; 6: e17745 21445232 10.1371/journal.pone.0017745 PMC3060165

[64] Furukawa S, Kawasaki Y, Miyamoto M, Hiyoshi M, Kitayama J, Akiyama T. The miR-1-NOTCH3-Asef pathway is important for colorectal tumor cell migration. PLoS One. 2013; 8: e80609 24244701 10.1371/journal.pone.0080609 PMC3823710

[65] Derrien T, Johnson R, Bussotti G, Tanzer A, Djebali S, Tilgner H, et al The GENCODE v7 catalog of human long noncoding RNAs: analysis of their gene structure, evolution, and expression. Genome Res. 2012; 22: 1775–1789. 22955988 10.1101/gr.132159.111 PMC3431493

[66] Barsyte-Lovejoy D, Lau SK, Boutros PC, Khosravi F, Jurisica I, Andrulis IL, et al The c-Myc oncogene directly induces the H19 noncoding RNA by allele-specific binding to potentiate tumorigenesis. Cancer Res. 2006; 66: 5330–5337. 16707459 10.1158/0008-5472.CAN-06-0037

